# Smoking cessation advice recorded during pregnancy in United Kingdom primary care

**DOI:** 10.1186/1471-2296-15-21

**Published:** 2014-02-01

**Authors:** Bethany Hardy, Lisa Szatkowski, Laila J Tata, Tim Coleman, Nafeesa N Dhalwani

**Affiliations:** 1 Division of Epidemiology and Public Health, University of Nottingham, Clinical Sciences Building, Nottingham City Hospital, Hucknall Road, Nottingham NG5 1 PB, UK; 2 Division of Primary Care, University of Nottingham, Queen’s Medical Centre, Nottingham NG7 2UH, UK

**Keywords:** Pregnancy, Smoking, Primary care, Smoking cessation advice

## Abstract

**Background:**

United Kingdom (UK) national guidelines recommend that all pregnant women who smoke should be advised to quit at every available opportunity, and brief cessation advice is an efficient and cost-effective means to increase quit rates. The Quality and Outcomes Framework (QOF) implemented in 2004 requires general practitioners to document their delivery of smoking cessation advice in patient records. However, no specific targets have been set in QOF for the recording of this advice in pregnant women. We used a large electronic primary care database from the UK to quantify the pregnancies in which women who smoked were recorded to have been given smoking cessation advice, and the associated maternal characteristics.

**Methods:**

Using The Health Improvement Network database we calculated annual proportions of pregnant smokers between 2000 and 2009 with cessation advice documented in their medical records during pregnancy. Logistic regression was used to assess variation in the recording of cessation advice with maternal characteristics.

**Results:**

Among 45,296 pregnancies in women who smoked, recorded cessation advice increased from 7% in 2000 to 37% in 2004 when the QOF was introduced and reduced slightly to 30% in 2009. Pregnant smokers from the youngest age group (15–19) were 21% more likely to have a record of cessation advice compared to pregnant smokers aged 25–29 (OR 1.21, 95% CI 1.10-1.35) and pregnant smokers from the most deprived group were 38% more likely to have a record for cessation advice compared to pregnant smokers from the least deprived group (OR 1.38, 95% CI 1.14-1.68). Pregnant smokers with asthma were twice as likely to have documentation of cessation advice in their primary care records compared to pregnant smokers without asthma (OR 1.97, 95% CI 1.80-2.16). Presence of comorbidities such as diabetes, hypertension and mental illness also increased the likelihood of having smoking cessation advice recorded. No marked variations were observed in the recording of cessation advice with body mass index.

**Conclusion:**

Recorded delivery of smoking cessation advice for pregnant smokers in primary care has increased with some fluctuation over the years, especially after the implementation of the QOF, and varies with maternal characteristics.

## Background

Smoking during pregnancy is harmful to both the mother and the unborn child and is associated with substantial morbidities such as ectopic pregnancy, premature rupture of membranes, pre-eclampsia, placental abruption, stillbirth, low birth weight, premature birth and childhood asthma [1-5]. Data from the 2010 Infant Feeding Survey show that 26% of mothers in the United Kingdom (UK) smoked at some point before or during their pregnancy and 12% of women smoked throughout their pregnancy [6]. Given the high proportion of mothers currently smoking during pregnancy and the resulting health impacts, reducing smoking during pregnancy in the UK is a national priority [7].

Offering smokers brief cessation advice lasting no more than five minutes during routine consultations with a general practitioner (GP), during which doctors make clear that smoking is harmful and offer help with cessation [8], is one of the simplest and most cost-effective tools to reduce the burden of smoking in the general population and increases rates of quitting by two-thirds compared to unassisted quit rates of 4% (OR 1.66, 95% CI 1.42-1.94) [9]. In pregnant women, cessation rates with brief advice have been low (5-9%) compared with intense advice and counselling (14-17%) [10,11]. However, physician advice to quit has been cited by pregnant women as one of the most important factors which influences their decision to stop smoking [12] and has been recommended in the recent World Health Organsation guidance for the management of tobacco use in pregnancy [13]. Current UK guidelines also recommend that smoking cessation advice should be offered at every available opportunity by health professionals who come into contact with pregnant women, including GPs and midwives, as only after smoking and smoking cessation is raised can it be possible to refer women on for the more intensive behavioural support or other smoking cessation therapies that are known to work [14-17]. The Quality and Outcomes Framework (QOF) introduced in UK primary care in 2004 financially rewards GPs for offering cessation advice to smokers and documenting this advice in the patients’ electronic medical records [18]. However, there are no specific QOF targets for offering and recording cessation advice to pregnant women who smoke and little is known about the frequency with which smoking cessation advice is indeed routinely delivered and recorded by primary care health professionals during pregnancy. Data from Health Education Authority (HEA) surveys carried out in the 1990s showed that less than half the women interviewed who were smokers received cessation advice from a health professional [19], and another study conducted in 200 antenatal clinics in Leicester, UK reported that only 34% of current smokers received advice from their GP, 19% from a midwife, 12% from an obstetrician, 9% from family and friends and 26% received no advice at all [20].

Given the national guidelines and the effectiveness of smoking cessation advice in increasing quit rates, we aimed to determine the proportion of pregnant smokers with smoking cessation advice recorded in their electronic primary care records in recent UK data. In addition, we aimed to investigate whether socioeconomic factors and women’s existing medical conditions in pregnancy were associated with this recording.

## Methods

### Data source and study population

The Health Improvement Network (THIN) is an electronic primary care database containing anonymised patient records from general practices across the UK [21]. THIN was set up by Cegedim Strategic Data (CSD) Medical Research UK, formerly known as Epidemiology and Pharmacology Information Core (EPIC) and provides data for research purposes. The University of Nottingham has a license to use data from EPIC, subject to approval from the Scientific Review Committee (SRC) which reviews the ethics and research protocol. Ethical approval for the study was obtained from the THIN Scientific Review Committee (reference number 11–047).

The version of THIN used for this study covered approximately 5.7% of the population and contained data from 495 practices with a nationally representative sample of women of reproductive age (defined here as aged 15–49 years) [21]. Fertility rates in THIN are very similar to national fertility rates [22] and the population prevalence of smoking recorded in THIN has been previously validated at both national and regional levels [23,24]. Our study population included all pregnancies recorded in THIN from 2000 to 2009 in women of reproductive age which resulted in either a live birth or a stillbirth, and where women were considered to be smokers during pregnancy. Women were defined as smokers if they had a Read code [25] indicating smoking recorded in their medical records or a drug code for nicotine replacement therapy (NRT) during their pregnancy, or, in the absence of recording during pregnancy, if their last recorded Read code in the 27 months prior to pregnancy indicated smoking as defined in more detail previously [26].

### Recording of smoking cessation and women’s characteristics

Our main outcome of interest was whether pregnant women identified as smokers had Read codes [25] for smoking cessation advice recorded in their THIN records during the period of their pregnancy. Code lists are available from the authors on request.

Data were also extracted on women’s age at the start of their pregnancy, socioeconomic deprivation as measured by quintiles of the Townsend Index of deprivation [27] based on their home postcode, body mass index (BMI) before their pregnancy and morbidities common in pregnancy for which the recording of smoking status has been specifically incentivised by the QOF (hypertension, diabetes, asthma, and mental illness which included depression, anxiety, bipolar disorder, schizophrenia and other psychoses), during pregnancy or within 27 months before conception in line with the QOF recording rules [28]. A summary variable was also created for the presence of at least one chronic condition out of the morbidities under study. Missing data for Townsend quintile and BMI were included in separate categories in the analyses.

### Statistical analysis

Across the whole study period, annual proportions of pregnant smokers with records of smoking cessation advice were calculated as the number of pregnancies among smokers with recorded smoking cessation advice divided by the total number of pregnancies among smokers who gave birth in that year.

**Figure 1:**
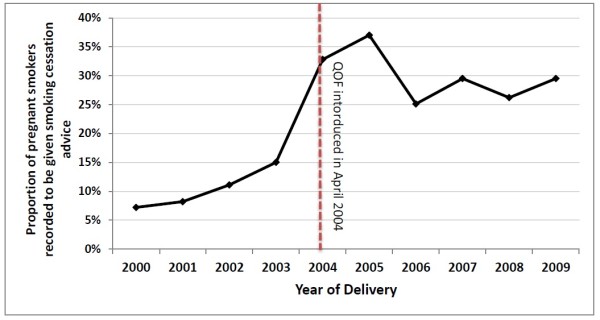
Annual proportions of pregnant smokers with smoking cessation advice recorded in their primary care records (2000–2009).

To investigate the factors associated with the recording of smoking cessation advice delivered to pregnant smokers we used data from 2006 to 2009, as the proportion of pregnant smokers given smoking cessation advice in primary care only stablised after 2006 (as seen in Figure 1). Firstly, using univariable logistic regression, odds ratios (ORs) and 95% confidence intervals (CIs) were calculated for the association between each variable (age at pregnancy, Townsend quintile, BMI category, asthma, diabetes, hypertension and mental illness) and whether or not smoking cessation advice was recorded during pregnancy. Covariates that were significantly associated with the recording of smoking cessation advice in the univariable model (p < 0.05) were considered for inclusion in the final multivariable model. As some women had more than one pregnancy during the study period that contributed to our analyses, we accounted for this potential clustering of pregnancies within women by calculating robust confidence intervals (CIs) around our odds ratios using the clustered sandwich estimator to allow for intragroup correlation [29,30]. All analyses were completed using Stata version 11.0 (StataCorp LP, College Station, TX).

## Results

### Baseline characteristics

We identified 45,296 pregnancies in 39,781 women resulting in a live birth or stillbirth from 2000 to 2009 and where women were classified as smokers during pregnancy. Of these 4,826 also had NRT prescribed during pregnancy for smoking cessation. The mean age at conception was 27 years (standard deviation 6.17) and 48.6% of the pregnancies included in the study were in women in the two most deprived quintiles of the Townsend Index of deprivation. Smoking cessation advice was recorded in 12,454 (27.5%) of all pregnancies under study and half of the pregnancies (49.5%) where women also received an NRT prescription during pregnancy. Table 1 describes the baseline characteristics of the study population.

**Table 1:** Baseline characteristics of the study population (pregnant smokers)

	**Total pregnancies (N = 45,296)**	Recorded smoking cessation advice (%*) (N = 12,454)	
**Age at conception**			
15-19 years	5,019	1,538	(30.6%)
20-24 years	12,180	3,355	(27.5%)
25-29 years	12,005	3,153	(26.3%)
30-34 years	9,736	2,613	(26.8%)
35-39 years	5,254	1,457	(27.7%)
40-44 years	1,048	317	(30.2%)
45-49 years	54	21	(38.9%)
**Townsend score in quintiles**			
Quintile 1 - most affluent	5,380	1,293	(24.0%)
Quintile 2	6,156	1,625	(26.4%)
Quintile 3	8,842	2,360	(26.7%)
Quintile 4	11,432	3,303	(28.9%)
Quintile 5 - most deprived	10,572	3,141	(29.7%)
Missing	5,380	1,293	(24.0%)
**Pre-conception body mass index**			
Normal (18.0-24.9)	19,579	5,144	(26.3%)
Underweight (<18.0)	2,106	588	(27.9%)
Overweight (25–29.9)	8,897	2,547	(28.4%)
Obese (> = 30)	6,338	1,874	(29.6%)
Missing	8,302	2,301	(27.7%)
**Asthma**	5,238	2,102	(40.1%)
**Hypertension**	969	315	(32.5%)
**Diabetes**	942	310	(32.9%)
**Mental illness**	7,193	2,184	(30.4%)
At least one of above morbidities**	12,577	4,177	(33.2%)

*% with recorded smoking cessation advice as a proportion of all pregnancies in smokers within each variable strata.

**Recording of medical conditions including asthma, hypertension, diabetes and mental illness.

### Annual trends in recorded smoking cessation advice in primary care

Figure 1 shows the annual proportions of pregnant smokers with smoking cessation advice recorded in their primary care medical records during pregnancy from 2000 to 2009. Overall, there has been an increase in this proportion over time. The proportion of pregnant smokers with recorded smoking cessation advice in 2000 was only 7%. This doubled to 15% in 2003, after which a steep increase was observed in 2004 with the proportion rising to 33%. The proportion of pregnant smokers with recorded smoking cessation advice peaked in 2005 at 37%, after which it stabalised at between 26-29% in the period of 2006–2009.

### Factors associated with the recording of smoking cessation advice in pregnancy

Table 2 shows variations in the odds of smoking cessation advice being recorded during pregnancy by women’s sociodemographic characteristics and morbidities. Pregnant smokers from the youngest age group (15–19) and the oldest age group (45–49) were more likely to be recorded as having received smoking cessation advice compared to pregnant smokers between the age of 25 and 29 years (OR 1.21 (95% CI 1.10-1.35) and OR 2.37 (95% CI 1.11-5.10) respectively). Recording also varied with socioeconomic status, such that pregnant smokers from the most deprived group (quintile 5) were 38% more likely to have smoking cessation advice recorded in their primary care records than pregnant women from the least deprived quintile (OR 1.38, 95% CI 1.14-1.68). In addition, recorded smoking cessation advice was higher in pregnant smokers with morbidities, such that pregnant smokers with asthma were almost twice as likely to have been recorded as having received smoking cessation advice compared to pregnant smokers without asthma (OR 1.97, 95% CI 1.80-2.16). Similarly, pregnant smokers with hypertension and diabetes were, respectively, 32% (OR 1.32, 95% CI 1.09-1.60) and 24% (OR 1.24, 95% CI 1.03-1.50) more likely to have smoking cessation advice recorded in their medical records compared to smokers without these morbidities. The presence of at least one of the above morbidities (diabetes, hypertension, asthma, mental illness) increased the likelihood of recording of smoking cessation advice for pregnant smokers by 49% (OR 1.49, 95% CI 1.39-1.60).

**Table 2:** Odds ratios of receiving smoking cessation advice by women’s characteristics and morbidities between 2006 and 2009

	**Pregnant smokers (n = 27,959)**	**Pregnant smokers with smoking cessation advice (n = 7,716)**		**Unadjusted**		**Adjusted**	
**Age at conception**		**n**	**%**	**OR (95% CI)**	**p-value**	**OR (95% CI)**	**p-value**
15–19	3,169	957	30.2	1.19 (1.08-1.32)	0.008	1.21 (1.10-1.35)	0.001
20–24	7,738	2,127	27.5	1.05 (0.96-1.14)		1.04 (0.96-1.13)	
25–29	7,542	2,006	26.6	1		1	
30–34	5,639	1,535	27.2	1.03 (0.95-1.12)		1.05 (0.96-1.14)	
35–39	3,166	872	27.5	1.05 (0.95-1.15)		1.07 (0.97-1.17)	
40–44	671	203	30.3	1.20 (1.00-1.43)		1.18 (0.98-1.41)	
45-49	34	16	47.1	2.45 (1.21-4.98)		2.37 (1.11-5.10)	
**Townsend score**							
Quintile 1 (most affluent)	3,047	711	23.3	1.00	<0.001*	1.00	<0.001*
Quintile 2	3,745	1,005	26.8	1.21 (1.07-1.35)		1.19 (1.06-1.34)	
Quintile 3	5,532	1,480	26.8	1.20 (1.06-1.36)		1.18 (1.04-1.35)	
Quintile 4	7,191	2,075	28.9	1.33 (1.16-1.53)		1.29 (1.13-1.48)	
Quintile 5 (most deprived)	6,583	1,989	30.2	1.42 (1.17-1.72)		1.38 (1.14-1.68)	
Missing	1,861	456	24.5	1.07 (0.89-1.28)		1.03 (0.85-1.24)	
**Body mass index**							
Underweight (<18.0)	11,893	3,196	26.9	1.10 (0.97-1.25)	<0.001	1.08 (0.95-1.22)	<0.001
Normal (18.0-24.9)	1,334	385	28.9	1		1	
Overweight (25.0-29.9)	5,689	1,645	28.9	1.11 (1.03-1.19)		1.09 (1.01-1.18)	
Obese (≥30)	4,218	1,252	29.7	1.15 (1.06-1.24)		1.08 (0.99-1.16)	
Missing	4,825	1,238	25.7	0.94 (0.87-1.01)		0.92 (0.83-1.01)	
**Asthma**	3,317	1,368	41.2	2.02 (1.85-2.2)	<0.001	1.97 (1.80 - 2.16)	<0.001
**Hypertension**	580	200	34.5	1.39 (1.16-1.67)	<0.001	1.32 (1.09 - 1.60)	<0.001
**Diabetes**	635	208	32.8	1.29 (1.07-1.55)	0.008	1.24 (1.03 - 1.50)	0.015
**Mental illness**	4,390	1,314	29.9	1.15 (1.06-1.24)	0.001	1.09 (1.01 - 1.18)	0.019

*OR* Odds ratio, *CI* Confidence interval, *p-value for trend.

## Discussion

Using a large population-based dataset, we have shown that the proportion of pregnant smokers recorded as having been advised to quit in primary care increased from 7% in 2000 to 30% in 2009, with substantial increases in the rate of recording around the time of the introduction of the QOF in 2004. We also found smoking cessation advice was more likely to be recorded in pregnant smokers from more deprived socioeconomic groups, among pregnant teenagers and women over age 45 years, and among women with asthma, diabetes, hypertension and mental illness.

Whilst national trends in the delivery of smoking cessation advice have been assessed in the general population [31,32], this is the first study to assess this advice recording during pregnancy in primary care. Our study provides estimates for the delivery of smoking cessation advice during pregnancy in routine GP consultations to complement survey data, which may over-estimate physician behaviours such as delivering smoking cessation advice [33] and may be limited by small sample sizes and non-probability sampling techniques [19,20]. However, we acknowledge that the recording of smoking cessation advice in a pregnant woman’s medical records may not always be acknowledged and interpreted as advice to quit by the women, and we do not know whether it was acted upon and resulted in a cessation attempt. The concept of smoking cessation advice is very subjective and different GPs may have different opinions on what constitutes effective advice. This may vary from a detailed discussion on smoking cessation strategies to only a brief mention of smoking during the consultation [34]. Indeed it is possible that in some cases smoking or smoking cessation may not actually have been discussed at all in the consultation and therefore we cannot be completely sure of the degree to which these Read codes represent the nature and extent of the advice delivered to pregnant smokers [32,34]. Additionally, GPs commonly address an average of two to three different medical problems during a single consultation [35,36]. However, the clinical coding does not necessarily reflect the breadth of the consultation and only the dominant topics of the visit may be coded [37]. Therefore, it is possible that smoking cessation advice was provided as part of the consultation yet not recorded electronically in women’s primary care notes. Furthermore, defining women as smokers based on NRT prescriptions may result in over-estimation of the cessation advice recording as prescribing of NRT is more likely to be accompanied or preceded by the delivery of smoking cessation advice. However, only 10% of the smokers in our study were identified based on NRT prescriptions. Moreover, only 50% of women who received NRT also had a record of smoking cessation advice, and therefore it would not affect the proportion of smokers with cessation advice substantially.

In the UK health care system midwives are the main point of contact for most women during pregnancy [37,38] and guidelines indicate that midwives should ask about women’s smoking status at the first antenatal booking appointment (usually between 8–12 weeks), and provide smoking cessation advice and referral if warranted [39]. This information should be documented in women’s hand-held notes (mandatory paper records that women should carry throughout pregnancy as part of the UK’s National Health Service antenatal care). However, there are no existing studies to show the extent to which this information is transferred to their electronic primary care records. We may, therefore, have underestimated the proportion of smokers in fact receiving cessation advice.

Our study is novel in that it investigates the maternal characteristics associated with the recording of smoking cessation advice during pregnancy. We found a significant increase in recorded smoking cessation advice with increasing deprivation quintile. A similar trend was seen in a study which examined the impact of the QOF on the recording of smoking advice in the general adult population - smokers from the most deprived quintile were 20% more likely to have a record of smoking cessation advice than smokers in the least deprived quintile [31]. This may be related to a poorer overall health status, higher prevalence of illness in more deprived smokers [40], or generally heavier smoking habits in this group [6], resulting in more GP visits and consequently more opportunities for the delivery and recording of smoking cessation advice. We also found that pregnant smokers in the youngest (15–19 years) and the oldest (45–49 years) age groups were more likely to have smoking cessation advice recorded during pregnancy. Although the latter was only a very small group of women, pregnancies in the 45–49 age groups are generally high-risk, resulting in more GP visits than normal pregnancies, which will make smoking cessation more important and result in more opportunities for providing smoking cessation advice. The prevalence of smoking during pregnancy is generally higher in younger women [6], and teenagers also have generally higher-risk pregnancies compared with women of average childbearing age [41,42]. According to the Infant Feeding Survey 2010, levels of smoking during pregnancy were the highest among mothers under the age of 20 in England and Scotland [6], which may explain higher smoking cessation advice documentation in this very young group in our study. The presence of comorbidities such as asthma, diabetes, hypertension and mental illness was also related to recording of smoking cessation advice delivery in our study. The effect of asthma was the strongest, such that pregnant smokers with asthma were twice as likely to have cessation advice recorded in their primary care records compared to non-asthmatics. This is consistent with a general population study which showed that presence of comorbidities was strongly related to the recording of cessation advice in primary care in the general population. However, the magnitude of effect for the morbidities was much higher than that found in our study [31], which may be because pregnant women are generally younger and healthier compared to the general adult population.

In our study, the proportion of pregnant smokers with smoking cessation advice recorded in their medical records during their pregnancy doubled between 2003 and 2004 suggesting that, despite having no specific target for recording of smoking cessation advice during pregnancy, the QOF has increased the occurrence of such activity. This marked increase between 2003 and 2004 can be attributed to the introduction of the 2004 GP contract as the negotiations for this contract started between 2002 and 2003 [43]. A general population study using primary care data from over 300 practices throughout the UK to assess the effect of the QOF on recording of smoking status and smoking cessation advice found that although rates of recording of smoking cessation advice in patients’ electronic medical records had been increasing gradually since the year 2000, the rate of improvement accelerated from 2003, with a 3-fold increase observed between the first quarter of 2003 and the same period in 2004, just before the introduction of the QOF (Risk Ratio (RR) 3.03, 95% CI 2.98-3.09) [44]. This may be evidence that historically GPs have not documented their delivery of smoking cessation advice in patients’ primary care records and after the introduction of QOF in 2004 the documentation of such advice improved. Data collected by semi-structured interviews in antenatal clinics at one UK hospital in the mid-1990s found that 34% of pregnant smokers reported receiving advice to quit from their GP [20]. Similarly, annual surveys between 1992 and 1999 conducted on pregnant women throughout England found that the proportion of pregnant smokers who received advice from a health professional ranged from 38%-55% [19]. Patient recall is known to be biased towards over-reporting in questions about smoking cessation advice [33,45], which may explain why estimates from these surveys are higher than our estimates from THIN data presented here. However, the large difference between the proportion of women with cessation advice recorded in THIN prior to 2004 and these survey estimates suggests that the introduction of the QOF may have resulted in an improvement in the recording of advice, which GPs were already giving but not documenting [34]. Despite these uncertainties in the interpretation of the data presented here, the observation that only approximately one-third of smokers have the delivery of cessation advice recorded in their primary care medical records suggests there is substantial room for improvement in the provision of this important health advice, particularly during pregnancy.

## Conclusions

In conclusion, although there are no specific targets to encourage GPs to deliver and document smoking cessation to pregnant women, the effects of smoking-related QOF targets in the general population appear to have increased the overall recording of smoking cessation advice during pregnancy as well with some fluctuations over the years. Pregnancy offers a strategic opportunity for health professionals to promote smoking cessation and motivate women to give up as women are generally more receptive to cessation interventions [46], therefore every opportunity to encourage smoking cessation should be seized by the health care professionals even if it is in the form of brief advice lasting less only a few minutes. The inclusion in the QOF of a target on smoking cessation advice specifically during pregnancy may result in the topic of smoking being raised more frequently, more advice being given and recorded and more pregnant smokers being referred on for specialist support with quitting smoking.

## Competing interests

The authors declare that they have no competing interests.

## Authors’ contributions

NND, LJT, TC and LS conceived the idea for the study and analyses, which was conducted using a dataset created under supervision of LJT of women in their potential childbearing years from The Health Improvement Network database. BH carried out the data management and analysis under supervision by NND and LS and wrote the first draft of the manuscript. LJT and TC provided interpretations at different stages of the project and helped to draft the manuscript. All authors read and approved full drafts and the final manuscript.

## Pre-publication history

The pre-publication history for this paper can be accessed here:

http://www.biomedcentral.com/1471-2296/15/21/prepub
